# Deciphering autosomal and X-linked genetic effects of early growth traits in Murciano-Granadina goats via a multivariate animal model

**DOI:** 10.1016/j.vas.2025.100553

**Published:** 2025-12-07

**Authors:** Jamshid Ehsaninia, Mohammad Bagher Zandi, Moein Taned, Alireza Bagheripour

**Affiliations:** a Department of Agriculture, Minab Higher Education Center, University of Hormozgan, Bandar Abbas, Iran; b Department of Animal Science, Faculty of Agriculture, University of Zanjan, Zanjan, Iran; c Ghaleh-Ganj dairy farm, Fajr Isfahan Agricultural and Livestock Farm, Isfahan, Iran

**Keywords:** Breeding values, Correlation, Genetic trend, Heritability, Maternal effects, Sex-chromosome

## Abstract

This study aimed to quantify autosomal and sex-linked genetic parameters for early growth traits in the Murciano-Granadina goat breed. Data were collected from a commercial dairy herd in Ghaleh-Ganj, southern Iran. A total of 19,582 records for birth weight (BWT) and 9157 records for weaning weight (WWT) were analyzed, covering animals born between 2016 and 2023, descended from 460 bucks and 5382 does. Live weight measurements were used to calculate preweaning growth rate (PWGR), preweaning Kleiber ratio (PWKR), and preweaning growth efficiency (PWGE). Environmental effects were evaluated using *aov* package in R, and the optimal multivariate model was constructed by combining the best-fitting univariate models for each trait. Genetic parameters were estimated using this optimal multivariate animal model and the average information restricted maximum likelihood algorithm in WOMBAT software. Estimated direct autosomal heritabilities ($h_{a}^{2}$) were 0.05 for BWT, 0.08 for WWT, 0.06 for PWGR and PWGE, and 0.09 for PWKR. Corresponding direct sex-linked heritabilities ($h_{s}^{2}$) were 0.03 for BWT, 0.02 for WWT, 0.01 for PWGR, and PWKR, and 0.04 for PWGE. Excluding sex-linked chromosomal effects from optimal model led to a 3–14 % increase in autosomal additive genetic variance and a 7–20 % increase in the residual variance. Autosomal genetic correlations (r_a_) ranged from -0.70 (BWT-PWGE) to 0.83 (PWKR-PWGR), while sex-linked genetic correlations (r_s_) ranged from -0.75 (BWT-PWGE) to 0.93 (WWT-PWGE). These findings highlight the relevance of accounting for sex-linked additive inheritance in genetic evaluations, supporting more informed selection decisions and contributing to improved genetic progress in Murciano-Granadina goats.

## Highlights

• Genetic parameters were estimated for growth traits in Murciano-Granadina goats.
• X-linked and autosomal additive effects were simultaneously modeled.
• Maternal effects showed a notable influence on early growth traits.
• X-linked heritability was low but not negligible for body weight traits.
• Genetic trends differed between autosomal and X-linked EBVs over years.

## 1 Introduction

Goats in Iran represent approximately 2.81 % of the global goat population, highlighting their importance in national livestock systems. As key species, they serve as multipurpose economic resources, largely due to their high adaptability to unfavorable climatic conditions and semiarid environments (FAO, 2023; Menchaca et al., 2024). Globally, goats are an integral part of livestock production systems in many regions, contributing to the supply of meat, milk, and fiber while providing crucial economic support to millions of small-scale and rural households (Meza-Herrera et al., 2024; Menéndez-Buxadera et al., 2024). In recent years, Iran has undertaken targeted genetic improvement initiatives aimed at improving the productivity and genetic potential of its indigenous goat populations. One such strategy involves the importation of high-yielding foreign breeds to be incorporated into national breeding programs, particularly in southern regions.

Since 2015, approximately 5000 Murciano-Granadina (MG) goats, a breed recognized for its high milk yield and adaptability, have been imported into the country by the private sector (Kaveh Baghbadorani et al., 2024). This initiative launched a genetic improvement program aimed at enhancing the productivity of local goat farming systems and strengthen the livelihoods of rural flock holders in these areas (Mokhtari et al., 2024). To achieve these objectives, this breeding program has used MG goats both as purebreds in specialized dairy herds and as crossbreds with native populations to develop composite genotypes that combine the superior productivity of MG goats with the environmental adaptability of local breeds (Mokhtari et al., 2024). These ongoing programs provide the foundation for evaluating the genetic architecture of economically important traits in MG goats under Iranian production conditions, which is the main objective of the present study.

Recent studies on the Murciano-Granadina population in the Ghaleh-Ganj herd have established fundamental genetic parameters for this population. Mokhtari et al. (2024) characterized the genetic architecture of early growth traits, identifying optimal models that incorporated both direct and maternal effects while reporting low heritability estimates (0.04–0.08) with positive genetic correlations. In a complementary investigation, Ehsani and Ghafouri Kesbi (2024) reported heritability estimates ranging from 0.05 to 0.11 for growth and efficiency traits, while also demonstrating significant maternal imprinting effects on birth.

Pre-weaning growth and efficiency-related traits are economically important traits in goat production, as they directly influence the profitability of the breeding enterprise (Dige et al., 2021). To use these traits effectively as tools in goat breeding, understanding the genetic and environmental parameters of these traits within a population is crucial (Gül et al., 2023). Genetic effects are conventionally partitioned into direct additive genetic and maternal additive genetic components. However, the potential contribution of sex-linked genetic variance, particularly that associated with the X chromosome, is often neglected in genetic evaluations. This oversight is notable because the X chromosome's large size and high gene density enable it to significantly influence phenotypic variation (Druet & Legarra, 2020). Therefore, disentangling X-linked additive genetic effects from autosomal effects is crucial for improving the accuracy of genetic parameter estimates.

Earlier studies have determined the role of sex-linked inheritance in the expression of body weight and growth traits in some livestock breeds such as sheep (Maraveni et al., 2018; Mohammadi et al., 2019; Noorian et al., 2020), goats (Zamani & Almasi, 2017; Latifi et al., 2020; Naderi & Latifi, 2023), and poultry (Kaviani et al., 2023). These results suggest that a non-negligible portion of the phenotypic variation for these kinds of traits can be attributed to additive genetic effects associated with the X chromosome. The inclusion of sex-linked genetic effects in mixed-model analyses therefore holds the potential to enhance the accuracy of selection and genetic evaluation (Larsen et al., 2014; Ghafouri-Kesbi & Eskandarinasab, 2024).

Although genetic parameters for growth and efficiency traits have been assessed in Murciano-Granadina goats, the partitioning of variance into autosomal and X-linked components has not been thoroughly addressed in this breed. Hence, the objective of the current study was to estimate autosomal and sex-linked (co)variance components and genetic parameters for early growth and efficiency-related traits in Murciano-Granadina goats through a multi-trait animal model. In addition, autosomal and X-linked additive genetic trends were assessed over time on the basis of predicted breeding values.

## 2 Materials and methods

### 2.1 Herd description and management system

Data were collected from a private Murciano-Granadina dairy goat herd located in Ghaleh-Ganj, Kerman Province, southern Iran (27°31′N, 57°52′E; 409 m above sea level). The goats in this study were managed under an intensive production system on a single commercial farm. The animals were grouped and housed according to age, sex, and health status to ensure optimal management and welfare. After birth, newborn kids were immediately weighed and ear-tagged, and their pedigree information, including dam and sire identification, date of birth, sex, type of birth, and birth weight was recorded. The kids remained with their mothers for approximately 10 days postpartum, after which they were separated by sex and relocated to age-appropriate housing. Weaning took place at approximately 80 days of age. Does were first mated at approximately 9 months of age, once they reached a minimum body weight of 28 kg. A mating ratio of 15 does per buck was applied. All animals received routine veterinary care, including antiparasitic treatments and vaccinations against key diseases such as enterotoxemia, foot-and-mouth disease, goat pox, and brucellosis.

### 2.2 Data structure and pedigree information

The present study analyzed the kidding records of Murciano-Granadina goats born between 2016 and 2023. The traits examined included birth weight (BWT), weaning weight (WWT), pre-weaning growth rate (PWGR), preweaning Kleiber ratio (PWKR), and preweaning growth efficiency (PWGE). The preweaning growth rate (PWGR, g/day) was calculated as the average daily gain (ADG) from birth to weaning as PWGR = [(WWT - BWT)/ weaning age] × 1000. The Kleiber ratio for the pre-weaning period (PWKR) is defined as the ratio of ADG (g/day) to metabolic weight at weaning (WWT^0.75^) according to Kleiber (Kleiber, 1947). Pre-weaning growth efficiency (PWGE, %) was calculated according to Ghafouri-Kesbi and Abbasi (Ghafouri-Kesbi & Abbasi, 2019) via the formula PWGE = [(WWT - BWT)/BWT] × 100.

A total of 19,582 newborn kids from 460 bucks and 5382 does were recorded for BWT, and 9157 kids from 402 bucks and 3609 does were recorded for WWT and pre-weaning efficiency traits. Errors in the pedigree, including repeated animals, animals registered as one of their parents, and loops present in the pedigree, were detected and corrected using CFC software (Sargolzaei et al., 2006). The pedigree comprised 24,569 animals spanning seven generations, with maximum and average depths of six and 1.73 generations, respectively. The population included 557 sires and 6980 dams, averaging approximately 80 sires and 1000 dams per generation. Analysis of 5335 full-sib families revealed variability in family sizes (mean: 2.07; range: 2–7). Pedigree completeness was high (89.30 %), with 21,930 animals having both parents identified. The base population contained 2456 founders (10.0 %), and inbreeding levels were minimal (mean ∼ 0.13 %; 609 inbred animals). Pedigree structure of the MG goats is summarized in Table 1.

**Table 1:** Pedigree structure of the Murciano-Granadina goats.

Parameter	Number
Total animals	24,569
Inbred animals	609
Base animals	2456
Non-base animals	22,113
Generations	7
Average pedigree depth	1.73
Sires	557
Progenies per sire	21,980
Dams	6980
Progenies per dam	22,063
Animals with progeny	7537
Animals without progeny	17,032
Sires per generation	80
Dams per generation	1000
Average full-sib family size	2.07
Average inbreeding coefficient in all animals	0.0013
Average inbreeding coefficient in the inbred animals	0.0519
Minimum of inbreeding coefficients	0.0039
Maximum of inbreeding coefficients	0.25

### 2.3 Statistical analysis

#### 2.3.1 Fixed and non-genetic effects

Data management and preprocessing were carried out using the *dplyr* package (Mendiburu, 2023) in R (R Core Team, 2024). An analysis of variance was conducted using the *aov* function in R to identify significant fixed effects influencing the studied traits. The Tukey-Kramer test was applied to compare group means. Only significant (*p*< 0.05) effects were included in the univariate and multi-trait animal models for genetic analyses. The following linear model was used to evaluate the influence of fixed effects on growth and efficiency-related traits:

$$y_{\text{ijklmno}}=\mu +BY_{i}+BM_{j}+S_{k}+BT_{l}+DA_{m}+P_{n}+e_{\text{ijklmno}}$$

where y represents the observations for growth traits, *µ* is the overall mean, BY denotes the effect of birth year (*I*= 2016, …, 2023), BM is the effect of birth month (*j*= 1, …, 12), S is the fixed effect of sex (*k*= 1, 2), BT is the effect of birth type (*m*= 1, 2, 3), DA is the fixed effect of dam age at kidding (*l*= 1, …, ≥ 7), P is the fixed effect of parity (*n*= 1, … ,7), and e represents the random residual effect. Prior to the analysis, data quality control procedures were implemented. Records with missing or invalid IDs were removed. Outlier detection was performed based on mean ± 3 standard deviations for each trait. The normality of each trait was assessed using the skewness and kurtosis coefficients combined with visual inspection of diagnostic plots in R software. For comprehensive data quality assessment, outlier detection, and validation of statistical modeling assumptions, trait distributions were visualized through histograms and boxplots, provided as Supplementary Figures S1–S5. A heatmap of the correlation matrices was generated using the *corrplot* package (Wei & Simko, 2024) in R.

#### 2.3.2 Estimation of genetic parameters

Variance components and genetic parameters for all traits were estimated using the average information restricted maximum likelihood (AI-REML) algorithm implemented in WOMBAT (Meyer, 2013). The animal model included previously identified significant fixed effects and the following random effects: autosomal additive genetic (AAG), X-linked additive genetic (SLAG), maternal additive genetic (MGE), maternal permanent environmental (MPE), and residual effects.

##### 2.3.2.1 Model selection strategy

To ensure robust model specification and minimize potential bias from over-parameterization, a comprehensive model selection procedure was undertaken. Initially, six alternative univariate animal models were fitted for each trait, varying in their combinations of random effects (AAG, SLAG, MGE, MPE) and the inclusion of covariance between direct and maternal genetic effects (refer to Supplementary Tables S1–S3 for detailed results). Model comparison was performed using Akaike’s Information Criterion (AIC) (Akaike, 1974), calculated as AICᵢ = −2LogL_i_ + 2P_i_, where LogLᵢ represents the maximized log-likelihood of model i at convergence and P_i_ denotes the number of estimated (co)variance parameters. The model with the lowest AIC value for each trait was identified as the best-fitting univariate structure, which subsequently informed the construction of the multivariate framework.

##### 2.3.2.2 Multivariate model specification

The final multivariate model was constructed based on the trait-specific optimal random-effect structures identified in the preceding univariate analyses. This strategy ensured that a uniform variance–covariance structure was not imposed across all traits. Instead, each trait contributed to the multivariate system according to its own parsimonious and best-fitting configuration, thereby enhancing the biological relevance and statistical accuracy of the estimated parameters. The final multivariate model, referred to as the optimal model (Model 1) was specified as follows:

$$y_{i}=\;X_{i}b_{i}+\;Z_{ai}a_{i}+\;Z_{\mathrm{si}}s_{i}+\;Z_{\mathrm{mi}}m_{i}+Z_{\mathrm{pei}}pe_{i}+\;e_{i}\;\text{with}\;\text{Cov}\;\left(a,m\right)=0$$

where y_i_ is the vector of observations in the *i*th trait; **b_i_, a_i_, s_i_, m_i_, pe_i_**, and **e_i_** represent vectors of fixed, direct additive genetic in autosomal chromosomes, direct additive genetic in sex-linked (X chromosomes), maternal additive genetic, maternal permanent environmental, and residual effects for trait i, respectively. The incidence matrices **X_i_, Z_ai_, Z_si_, Z_mi_**, and $Z_{\mathrm{pei}}$ link **b_i_, a_i_, s_i_, m_i_**, and **pe_i_** to y_i_. The (co)variance structure of the random effects was defined as:

$$\left[\begin{array}{c} \begin{array}{c} A⊗\Sigma_{a}\;\;\;\;0\\ 0\;\;\;\;S\;⊗\Sigma_{s} \end{array}\;\;\;\;\begin{array}{c} \;0\;\;\;\;\;0\;0\\ \;0\;\;\;\;\;\begin{array}{c} \;0\;\;\;\;\;0\; \end{array} \end{array}\\ \begin{array}{c} 0\;\;\;\;\;0\\ \begin{array}{c} 0\\ 0 \end{array}\;\;\;\;\;\begin{array}{c} 0\\ 0 \end{array} \end{array}\;\;\;\;\begin{array}{c} \;A⊗\Sigma_{m}\;\;\;\;\;0\begin{array}{c} \;\;\;\;\;\;0\; \end{array}\\ \begin{array}{c} 0\\ 0 \end{array}\;\;\;\;\;\begin{array}{c} \begin{array}{c} I⊗\Sigma_{\mathrm{pe}}\\ 0\; \end{array}\;\;\;\;\;\begin{array}{c} 0\;\\ I⊗\Sigma_{e} \end{array} \end{array} \end{array} \end{array}\right]$$

where A is the numerator relationship matrix for autosomal loci, S is the X-chromosomal relationship matrix whose elements are functions of co-ancestries between relative for X-chromosomal loci (Fernando and Grossman, 1990). The construction of S and its inverse obtained by algorithm developed by Fernando and Grossman (1990) and I is an identity matrix of appropriate dimension. $\Sigma_{a}$, $\Sigma_{s}$, $\Sigma_{m}$, $\Sigma_{\text{pe}}$, and $\Sigma_{e}$ are 5 × 5 covariance matrices containing the variances (diagonal elements) and covariances (off-diagonal elements) among traits for each random effect. All random effects were assumed to be normally distributed as $a∼\text{MVN}(0,A⊗\Sigma_{a})$, $s∼\text{MVN}(0,S⊗\Sigma_{s})$, $m∼\text{MVN}(0,A⊗\Sigma_{m})$, $\text{pe}∼\text{MVN}(0,I⊗\Sigma_{\text{pe}})$, and $e∼\text{MVN}(0,I⊗\Sigma_{e})$. Each Σ matrix represents the (co)variance structure across the five traits. For instance, the autosomal additive genetic covariance matrix ($\Sigma_{a}$) was structured as:

$$\Sigma_{a}=\left[\begin{array}{c} \begin{array}{c} \sigma_{a,1}^{2}\\ \sigma_{a,21} \end{array}\begin{array}{c} \sigma_{a,12}\\ \sigma_{a,2}^{2} \end{array}\begin{array}{c} \begin{array}{c} \cdots \sigma_{a,15} \end{array}\\ \begin{array}{c} \cdots \sigma_{a,25} \end{array} \end{array}\\ \vdots \vdots \begin{array}{c} \ddots \vdots \end{array}\\ \sigma_{a,51}\sigma_{a,52}\begin{array}{c} \cdots \sigma_{a,5}^{2} \end{array} \end{array}\right]$$

where diagonal elements ($\sigma_{a,1}^{2}$ to $\sigma_{a,5}^{2}$) represent autosomal additive genetic variances for each trait, and off-diagonal elements ($\sigma_{a,\text{ij}}$) represent autosomal additive genetic covariances between traits i and j. Only variance components identified as significant in the univariate analyses were retained within their respective Σ matrices, while non-significant components were constrained to zero. This trait-specific modeling ensured the final multivariate framework was both statistically sound and biologically interpretable.

##### 2.3.2.3 Sensitivity analysis and model robustness

To evaluate the robustness of parameter estimates, the final multivariate model (Model 1) was compared with three alternative specifications:

Model 2 (excluding sex-linked effects):

$$y_{i}=\;X_{i}b_{i}+\;Z_{ai}a_{i}+\;Z_{\mathrm{mi}}m_{i}+Z_{\mathrm{pei}}pe_{i}+\;e_{i}\;\text{Cov}\;\left(a,m\right)=0$$

Model 3 (excluding maternal effects):

$$y_{i}=\;X_{i}b_{i}+\;Z_{ai}a_{i}+\;Z_{\mathrm{si}}s_{i}+\;e_{i}\;\text{Cov}\;\left(a,m\right)=0$$

Model 4 (including Cov(a,m)≠0)

$$y_{i}=\;X_{i}b_{i}+\;Z_{ai}a_{i}+\;Z_{\mathrm{si}}s_{i}+\;Z_{\mathrm{mi}}m_{i}+Z_{\mathrm{pei}}pe_{i}+\;e_{i}\;\text{Cov}\;\left(a,m\right)\neq 0$$

Model comparisons were based on AIC, and log-likelihood values. This comparative approach ensured that the final parameter estimates were robust and that biological inferences concerning the roles of autosomal, sex-linked, and maternal effects were not artifacts of a single model specification.

#### 2.3.3 Estimation of genetic correlations

Autosomal additive genetic (r_a_) and sex-linked additive genetic (r_s_) correlations among BWT, WWT, PWGR, PWKR, and PWGE were also estimated using optimal five-trait analysis to quantify the relationships between traits based on the following formulas (Mrode, 2005):

Autosomal additive genetic correlation (r_a_)

$$r_{a}=\frac{\text{cov}\left(a_{x},a_{y}\right)}{\sqrt{\sigma_{\text{ax}}^{2}\times \sigma_{\text{ay}}^{2}}}$$

Sex-linked additive genetic correlation (r_s_)

$$r_{s}=\frac{\text{cov}\left(s_{x},s_{y}\right)}{\sqrt{\sigma_{\text{sx}}^{2}\times \sigma_{\text{sy}}^{2}}}$$

where $\text{cov}(a_{x},a_{y})$ and $\text{cov}(s_{x},s_{y})$ are the autosomal additive genetic and sex-linked additive genetic covariance between the X and Y traits, $\sigma_{\text{ax}}^{2}$ and $\sigma_{\text{sx}}^{2}$ are the autosomal and sex-linked additive genetic variances for trait X, and $\sigma_{\text{ay}}^{2}$ and $\sigma_{\text{sy}}^{2}$ are the autosomal and sex-linked additive genetic variances for trait Y.

#### 2.3.4 Autosomal and X-linked genetic trends

Based on the best-fitting multivariate model constructed according to the single-trait analyses, autosomal and X-linked additive genetic breeding values were predicted using the best linear unbiased prediction (BLUP) method implemented in the WOMBAT program. These predicted breeding values represent the estimated genetic merit of each animal attributable to autosomal and X-linked loci, respectively. Autosomal and X-linked additive genetic trends were then evaluated by regressing the predicted breeding values of animals on their birth date expressed as a continuous covariate, rather than discrete birth years, to achieve a more precise estimation of genetic change over time. The analysis was performed using the lm() function in R (Torres-Vázquez et al., 2009) according to the following linear model:

$$Y_{i}=\;b_{1}x_{i}+\;b_{0}+\varepsilon_{i}$$

where *Y*_i_ is the estimated breeding value of the *i*th animal; b_1_ is the regression coefficient representing the genetic trend per year; $x_{1}$ is the is the continuous birth date; b_0_ is the intercept; and, ε_i_ is the random error. This approach provides a smoother and more informative depiction of genetic trends over time, improving both the accuracy of the estimates and the interpretability of graphical visualizations.

## 3 Results

### 3.1 Descriptive statistics

Table 2 presents the data details for the Murciano-Granadina goats. The average number of records per doe ranged from 2.53 (WWT, PWGR, PWKR, and PWGE) to 3.64 (BWT), while bucks had values ranging from 22.78 to 42.57. The overall mean values for BWT, WWT, PWGR, PWKR, and PWGE were 2.58 ± 0.38 kg, 10.58 ± 2.01 kg, 102.83 ± 23.15 g/day, 18.12 ± 3.21, and 331.04 ± 88.25, respectively. The coefficients of variation for these traits were 14.73 %, 19.00 %, 22.51 %, 17.72 %, and 26.70 %, respectively. All traits exhibited acceptable distributional properties, with skewness (0.08–0.61) and kurtosis (2.37–3.05) values within ranges suitable for parametric statistical methods.

**Table 2:** Characteristics of data structure for early growth traits of the Murciano-Granadina goat breed.

Items	BWT (kg)	WWT (kg)	PWGR (g/d)	PWKR	PWGE
No. of records	19,343	9157	9157	9157	9157
No. of animals	22,159	10,119	10,119	10,119	10,119
No. of sires	460	402	402	402	402
No. of dams	5382	3609	3609	3609	3609
No. of records per sire	42.57	22.78	22.78	22.78	22.78
No. of records per dam	3.64	2.53	2.53	2.53	2.53
Mean	2.58	10.58	102.83	18.12	331.04
SD	0.38	2.01	23.15	3.21	88.25
CV ( %)	14.73	19.00	22.51	17.72	26.70
Minimum	1.10	5.20	31.18	7.38	105.71
Maximum	4.20	20.20	334.13	32.47	983.33

BWT: birth weight, WWT: weaning weight, PWGR: preweaning growth rate, PWKR: preweaning Kleiber ratio, PWGE: preweaning growth efficiency, SD: standard deviation, CV: coefficient of variation.

### 3.2 Fixed effects

Table 3 presents the least square means and standard errors for the growth traits. While the trends varied, the year of birth significantly influenced (*p*< 0.01) all growth and efficiency-related traits. Kids born in 2019 presented the highest values for all traits except BWT, whereas the highest value for this trait was recorded in 2016. The birth month of the kids also significantly affected (*p*< 0.01) all the studied traits. Additionally, the sex of the kids was another significant source of variation (*p*< 0.01), with male lambs outperforming their female counterparts. Males were generally heavier at both birth and weaning. Birth type significantly affected all growth traits (*p*< 0.01), except for PWGR, with single-born kids showing superior performance compared with those born in multiples. The age of the doe significantly influenced all traits considered traits of the goats (*p*< 0.01). The effect of dam parity was significant (*p*< 0.05) for all traits, except for PWGR.

**Table 3:** Least squares mean (± standard error) for growth and efficiency-related traits of the Murciano-Granadina goat.

Fixed effects		Traits			
	BWT (kg)	WWT (kg)	PWGR (g/day)	PWKR	PWGE
Birth year	**	**	**	**	**
2016	2.29±0.060^d^	10.03±0.041^b^	99.69±0.407^c^	17.42±0.058^b^	310.09±2.072^c^
2017	2.32±0.007^d^	10.36±0.041^a^	102.01±0.616^b^	17.47±0.061^b^	329.54±2.041^bc^
2018	2.51±0.007^b^	10.47±0.031^a^	103.10±0.474^b^	17.66±0.062^b^	345.10±2.691^a^
2019	2.55±0.007^a^	10.52±0.032^a^	106.82±0.467^a^	18.38±0.078^a^	354.11±2.691^a^
2020	2.39±0.006^c^	10.15±0.027^b^	94.15±0.687^c^	16.17±0.064^c^	334.42±1.776^b^
2021	2.42±0.004^c^	10.10±0.046^b^	101.16±0.487^bc^	18.31±0.089^a^	318.76±2.998^c^
2022	2.49±0.005^ab^	10.00±0.059^b^	98.96±0.712^bc^	17.44±0.063^b^	316.34±3.560^c^
2023	2.50±0.007^ab^	9.79±0.112^c^	99.42±2.212^bc^	17.48±0.068^b^	314.29±13.560^c^
Gender	**	**	**	**	**
Male	2.55±0.005^a^	10.49±0.021^a^	106.29±0.321^a^	18.29±0.036^a^	341.09±1.371^a^
Female	2.33±0.005^b^	10.02±0.021^b^	95.85±0.313^b^	16.96± 0.037^b^	322.32±1.334^b^
Birth type	**	*	NS	*	**
Single	2.60±0.003^a^	10.35±0.023^a^	100.38±0.351	17.38±0.054^b^	319.22±1.531^a^
Twin	2.49±0.003^b^	10.28±0.020^a^	101.51±0.303	17.69±0.062^a^	337.06±1.321^b^
Triple	2.33±0.017^c^	10.04±0.070^b^	98.82± 1.01	17.53±0.155^ab^	364.79±4.588^a^
Parity	**	*	NS	**	**
1	2.43±0.011^abc^	9.09±0.029^c^	100.20±0.304	16.74±0.089^d^	319.14±1.988^c^
2	2.41±0.008^c^	9.73±0.034^b^	100.63±0.211	16.88±0.056^d^	321.17±1.129^bc^
3	2.42±0.007^bc^	10.39±0.029^b^	101.01±0.672	17.37±0.038^bc^	323.14±2.118^b^
4	2.43±0.007^abc^	10.42±0.028^a^	100.54±0.513	17.49±0.089^ab^	327.84±3.745^a^
5	2.45±0.007^a^	10.29±0.059^c^	99.87±0.456	17.51±0.144^a^	321.44±2.444^bc^
6	2.44±0.006^ab^	10.41±0.045^bc^	99.65±1.489	17.40±0.105^c^	322.19±3.314^bc^
7	2.42±0.013^abc^	10.40±0.124^bc^	98.96±2.005	17.01±0.185^d^	314.89±8.200^d^
Age of doe (year)	**	**	**	*	**
1	2.29±0.006^c^	10.02±0.034^c^	101.81±0.507^b^	17.28±0.075^c^	317.08±2.951^c^
2	2.47±0.005^b^	10.34±0.034^b^	104.63±0.416^a^	17.58±0.065^b^	323.17±2.240^c^
3	2.53±0.006^a^	10.31±0.029^b^	101.11±0.447^b^	17.65±0.074^ab^	336.25±1.817^b^
4	2.45±0.007^b^	10.53±0.028^a^	97.26±0.513^c^	17.85±0.061^a^	354.06±2.214^a^
5	2.46±0.010^b^	9.97±0.059^c^	94.27±0.874^c^	17.29±0.073^c^	316.56±3.815^c^
6	2.50±0.025^a^	10.28±0.045^bc^	95.55±1.121^c^	17.31±0.128^c^	317.12±4.257^c^
7	2.44±0.028^b^	10.24±0.124^bc^	92.28±2.629^c^	16.86±0.382^d^	316.12±11.482^c^
Birth month	**	**	**	**	**

BWT: birth weight, WWT: weaning weight, PWGR: preweaning growth rate, PWKR: preweaning Kleiber ratio, PWGE: preweaning growth efficiency, *: *p<* 0.05, **: *p<* 0.01, NS: Not significant.

^a,b,c^ Means with different letters within the same column and subclass are significantly different.

### 3.3 Model selection and fit criteria

Univariate analyses were first conducted for each trait to identify the best-fitting random effect structures, informed by Akaike’s Information Criterion (AIC). These preliminary analyses revealed that the optimal model for BWT included autosomal additive genetic (AAG), sex-linked additive genetic (SLAG), maternal genetic (MGE), and maternal permanent environmental (MPE) effects, with the direct–maternal genetic covariance constrained to zero (model 5, Supplementary Table S1). For WWT and the efficiency traits (PWKR, PWGR, PWGE), the best-fitting univariate models retained SLAG and MGE effects, also with Cov(a,m) = 0 (model 3, Supplementary Tables S2-S3). The final multivariate model was constructed using these trait-specific optimal structures, thereby avoiding the imposition of a uniform and potentially suboptimal variance-covariance structure across all traits.

### 3.4 Multivariate model and sensitivity analysis

Random effect, log-likelihood, and Akaike information criterion (AIC) values for growth traits in Murciano-Granadina goat under different multivariate animal models are presented in Table 4. Model selection based on AIC identified Model 1, which included autosomal additive genetic (AAG), sex-linked additive genetic (SLAG), maternal additive genetic (MGE), and maternal permanent environmental (MPE) effects, as the optimal model (AIC = 84,423.06). Excluding SLAG effects (Model 2) resulted in an increase of +28.90 in AIC and systematic alterations in variance components, including an increase in autosomal additive genetic variance ($\sigma_{a}^{2}$: 12.4–14.3 % for BWT, WWT, and PWGE) accompanied by substantial reductions in maternal genetic variance ($\sigma_{m}^{2}$: 77.0 % to 79.0 % for WWT and PWGE). Omitting maternal effects (Model 3) caused more pronounced distortions (ΔAIC = +47.66), with substantial inflation in both autosomal additive genetic variance ($\sigma_{a}^{2}$: 45.2–514.3 % across traits) and sex-linked variance (25.0–140.3 %). Model 4, which estimated direct–maternal genetic covariance, exhibited the poorest model fit (ΔAIC = +210.62) and yielded biologically implausible correlation estimates (r_a,m:_ −0.79 ± 0.14 to 0.49 ± 0.21) characterized by high standard errors relative to parameter estimates.

**Table 4:** Random effect, LogL and AIC values for growth traits in Murciano-Granadina goat under different multivariate animal models.

Model	Random effect	Log L	AIC
Model 1	AAG, SLAG, MTG, MPE	−42,150.53	84,423.06
Model 2	AAG, MTG, MPE	−42,160.51	84,451.96
Model 3	AAG, SLAG	−42,190.36	84,470.72
Model 4	AAG, SLAG, MTG, MPE	−42,230.84	84,633.68

AAG: autosomal additive genetic effect, SLAG: sex-linked additive genetic effect, MPE: maternal permanent environmental effect, MTG: maternal additive genetic effect, AIC: Akaike information criteria.

### 3.5 Maternal effects

Table 5 present estimates of variance components and genetic parameters for the studied traits. Maternal heritability estimates ($h_{m}^{2}$) from the optimal model demonstrated notable trait-specific patterns, ranging from 0.04 ± 0.02 for WWT to 0.13 ± 0.02 for BWT (Table 5). The exclusion of maternal effects substantially deteriorated model fit (ΔAIC = +47.66) and led to inflated $h_{a}^{2}$ estimates across all traits. For BWT, $\sigma_{a}^{2}$ increased more than sixfold and $h_{a}^{2}$ rose from 0.05 to 0.30 when maternal effects were omitted, demonstrating significant confounding between direct and maternal genetic effects. Maternal permanent environmental effects (pe^2^) were particularly important for birth weight but negligible for other traits.

**Table 5:** Estimates of variance components and genetic parameters from different multivariate model of early growth traits in Murciano-Granadina goats.

**Model**	**Trait**	$\sigma_{a}^{2}$	$\sigma_{s}^{2}$	$\sigma_{m}^{2}$	$\sigma_{\mathrm{pe}}^{2}$	$\sigma_{a,m}$	$\sigma_{e}^{2}$	$\sigma_{P}^{2}$	$h_{a}^{2}$**±SE**	$h_{s}^{2}$**±SE**	$pe^{2}$**±SE**	$h_{m}^{2}$**±SE**	$r_{a,m}$
1	BWT	0.007	0.004	0.018	0.001		0.108	0.138	0.05±0.01	0.03±0.01	0.01±0.02	0.13±0.02	-
	WWT	0.126	0.036	0.061	-		1.430	1.654	0.08±0.02	0.02±0.01	-	0.04±0.02	-
	PWGR	22.96	5.18	20.15	-		320.80	369.09	0.06±0.02	0.01±0.01	-	0.05±0.02	-
	PWKR	0.676	0.062	0.370	-		6.70	7.81	0.09±0.02	0.01±0.01	-	0.05±0.02	-
	PWGE	440.41	291.24	589.06	-		5885.20	7205.90	0.06±0.02	0.04±0.01	-	0.08±0.02	-
2	BWT	0.007	-	0.004	0.018		0.108	0.138	0.05±0.01	-	0.13±0.01	0.03±0.01	-
	WWT	0.128	-	0.014	0.074		1.427	1.643	0.08±0.02	-	0.05±0.02	0.01±0.02	-
	PWGR	22.56	-	15.29	7.38		321.86	367.09	0.06±0.02	-	0.02±0.03	0.04±0.03	-
	PWKR	0.653	-	0.303	0.119		6.713	7.788	0.08±0.02	-	0.02±0.03	0.04±0.02	-
	PWGE	480.18	-	123.49	617.47		5881.22	7288.00	0.07±0.02	-	0.09±0.02	0.02±0.02	-
3	BWT	0.043	0.005	-	-	-	0.095	0.143	0.30±0.02	0.03±0.01	-	-	-
	WWT	0.183	0.042	-	-	-	1.44	1.66	0.11±0.02	0.03±0.01	-	-	-
	PWGR	43.00	7.80	-	-	-	321.23	372.03	0.12±0.02	0.02±0.01	-	-	-
	PWKR	1.02	0.149	-	-	-	6.70	7.88	0.13±0.02	0.02±0.02	-	-	-
	PWGE	1388.88	365.75	-	-	-	5624.71	7379.31	0.19±0.02	0.05±0.01	-	-	-
4	BWT	0.007	0.003	0.019	0.001	−0.001	0.110	0.140	0.05±0.01	0.02±0.01	0.01±0.01	0.11±0.02	−0.34±0.23
	WWT	0.173	0.029	0.186	-	−0.012	1.404	1.650	0.11±0.03	0.02±0.01	-	0.11±0.03	−0.79±0.14
	PWGR	26.07	5.37	38.15	-	0.08	318.37	368.37	0.07±0.02	0.02±0.01	-	0.10±0.02	0.04±0.27
	PWKR	0.915	0.086	0.824	-	0.03	6.55	7.82	0.12±0.03	0.01±0.01	-	0.10±0.02	0.49±0.21
	PWGE	454.91	238.48	731.55	-	−0.30	5891.36	7167.12	0.06±0.02	0.02±0.01	-	0.10±0.03	0.39±0.22

BWT: birth weight, WWT: weaning weight, PWGR: preweaning growth rate, PWKR: preweaning Kleiber ratio, PWGE: preweaning growth efficiency, $\sigma_{a}^{2}$: additive genetic variance for autosomal loci, $\sigma_{s}^{2}$: additive genetic variance for sex-linked loci, $\sigma_{m}^{2}$: maternal additive genetic variance, $\sigma_{\text{pe}}^{2}$: maternal permanent environmental variance, $\sigma_{a,m}$: direct-maternal additive genetic covariance, $\sigma_{e}^{2}$: residual variance, $\sigma_{P}^{2}$: phenotypic variance, $h_{a}^{2}$: direct autosomal heritability, $h_{s}^{2}$: direct sex-linked heritability, $pe^{2}$: maternal permanent environmental effect, $h_{m}^{2}$: maternal heritability, $r_{a,m}$: direct-maternal additive genetic correlation, SE: standard error.

### 3.6 Variance component and heritability estimates

Including the effects of the sex-linked chromosome in the model led to a reduction in direct genetic variance of 3 %, 11 %, 12 %, 14 %, and 13 % for the traits PWKR, PWGR, PWGE, BWT and WWT, respectively. Based on our best-fitting model, the proportion of sex-linked variance ranged from 8 % to 40 % across traits, with the highest value observed PWGE. The best model revealed a trend of increasing autosomal variances from BWT to WWT. The direct autosomal heritabilities ($h_{a}^{2}$) were 0.05 ± 0.01 for BWT, 0.08 ± 0.02 for WWT, 0.06 ± 0.02 for PWGR, 0.09 ± 0.02 for PWKR, and 0.06 ± 0.02 for PWGE. In contrast, the corresponding direct sex-linked heritability estimates ($h_{s}^{2}$) were relatively low: 0.03 ± 0.01 for BWT, 0.02 ± 0.01for WWT, 0.01 ± 0.01 for PWGR, 0.01 ± 0.01 for PWKR, and 0.04 ± 0.01 for PWGE. Maternal heritability ($h_{m}^{2}$) were 0.13 ± 0.02, 0.04 ± 0.02, 0.05 ± 0.02, 0.05 ± 0.02, and 0.08 ± 0.02 for BWT, WWT, PWGR, PWKR, PWGE, respectively.

### 3.7 Autosomal and sex-linked additive genetic correlations

**Fig. 1:**
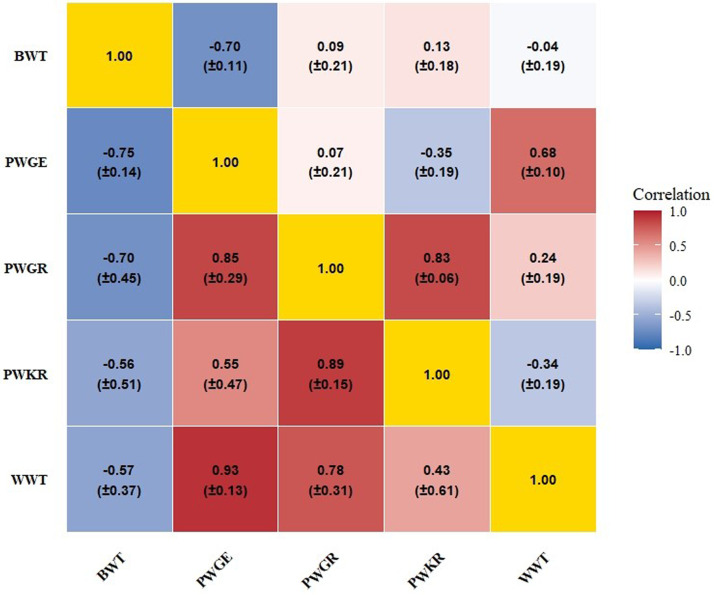
Autosomal additive genetic (below the diagonal) and sex-linked additive genetic (above the diagonal) correlations between growth and efficiency traits in Murciano-Granadina goats.

Estimates of autosomal (rₐ) and sex-linked additive genetic correlations (rₛ) among early growth traits are presented in Fig. 1. Autosomal correlations (rₐ) ranged from −0.70 (BWT-PWGE) to 0.83 (PWGR-PWKR), indicating moderate to strong associations for specific trait pairs. Positive rₐ values were observed between WWT and PWGE (0.68) and WWT and PWGR (0.24), whereas correlations between BWT and later growth traits were generally low or slightly negative (−0.04 to 0.13). Sex-linked correlations (rₛ) exhibited a broader range (−0.75 to 0.93), reflecting a more variable contribution of X-linked loci across traits. Strong negative rₛ correlations were detected between BWT and post-weaning traits, including PWGE (−0.75), PWGR (−0.70), and PWKR (−0.56). In contrast, robust positive correlations were observed among post-weaning traits, notably WWT-PWGE (0.93), PWGR-PWGE (0.89), and PWGR-PWKR (0.89).

### 3.8 Breeding values and genetic trends

Descriptive statistics of autosomal and sex-linked additive genetic estimated breeding values (EBVs) for pre-weaning growth traits are summarized in Supplementary Table S4. Across all traits, mean autosomal EBVs were positive, reflecting favorable genetic potential, with values such as those for BWT (0.003) and PWKR (0.001) showing minimal deviations from zero. In contrast, mean sex-linked additive genetic EBVs were generally lower in magnitude, often near zero or slightly negative, indicating a relatively limited contribution of X-linked loci to early growth traits. The range and standard deviations of the EBVs revealed substantial individual variability, particularly for traits with higher phenotypic variance such as PWGR and PWGE, suggesting potential for selection based on both autosomal and sex-linked additive genetic effects.

**Fig. 2:**
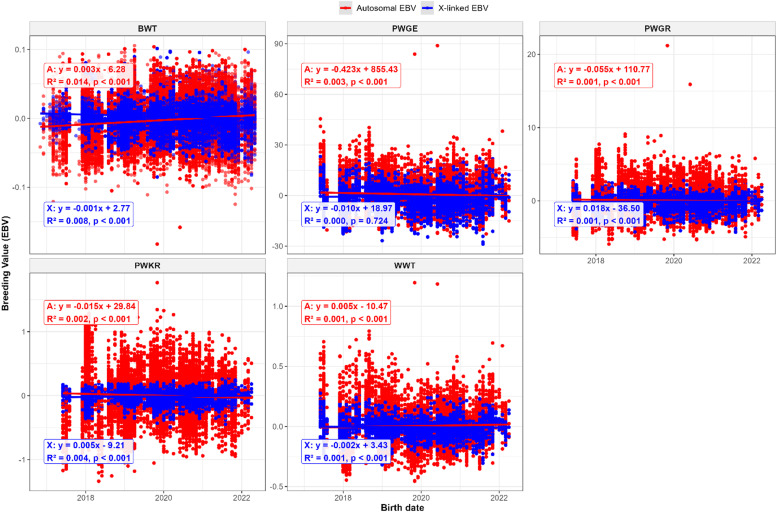
Comparison of autosomal and X-linked additive genetic trends for different growth traits in Murciano-Granadina goats, utilizing continuous birth date analysis.

Fig. 2 illustrate the genetic trends of the studied traits based on autosomal and sex-linked additive genetic EBVs over birth dates. Autosomal additive genetic trends were positive for BWT (0.003 kg/year) and WWT )0.005 kg/year). Conversely, PWGR (−0.055 kg/year), PWKR (−0.015 units/year), and PWGE (−0.423 units/year) exhibited negative autosomal trends. For sex-linked additive effects, BWT (−0.001 kg/year), WWT (−0.002 kg/year), and PWGE (−0.010 units/year) showed slight negative annual trends, whereas PWGR (0.018 kg/year) and PWGE (0.005 units/year) displayed positive trends.

## 4 Discussions

### 4.1 Environmental effects

The coefficient of variation for all growth traits varied from 14.73 % to 26.70 %, indicating substantial phenotypic variability; hence, these traits can be improved by selection procedures or by providing better environmental conditions, as described by Gautam et al. (2019). Kids born in 2018 and 2019 showed superior performance across all evaluated traits. The variation in climatic conditions, milk-feeding system for kids, and disease incidence may account for the variability observed among different years and months (Gupta et al., 2016). Compared with female kids, male kids presented increased performance across all the measured traits. This trend is consistent with previous findings (Gül et al., 2023; Tesema et al., 2024; Wang et al., 2024) and may be explained by hormonal mechanisms, particularly the inhibitory effect of estrogen on long bone growth in females (Balasundaram et al., 2023; Besufkad et al., 2024). Single-born kids exhibited higher birth and weaning weights compared to twins and triplets, which is consistent with results from Kilis (Gül et al., 2023) and other goat breeds (Mohammed et al., 2018; Dige et al., 2021).

However, for efficiency traits such as PWKR and PWGE, triplet-born kids demonstrated superior performance, likely due to compensatory adaptations resulting from intra- and postnatal competition for limited maternal resources (Mokhtari et al., 2019; Tesema et al., 2021). Additionally, efficiency-traits improved with increasing dam age, reaching their highest point at about six years, followed by a notable decline. The pattern is in agreement with earlier studies (Zhang et al., 2009; Latifi et al., 2020; Tesema et al., 2020) and may reflect the maturity of the dams and their potential for milk production at intermediate ages. Parity had significant effect (*p*< 0.01) on BWT of the kids. The results indicated that kid BWT was lowest at the first parity and increased significantly up to the fifth parity, after which it declined toward the seventh parity, which contradicts the findings of Alphonsus et al. (2010) in Sokoto goats. Since does do not attain full physiological maturity during their first reproductive cycle, the observed increase in kid birth weight with advancing parity is considered a natural phenomenon. Parity had no significant effect on PWGR; however, some studies have reported an increase in PWGR with higher parity (Lamesegn et al., 2018; Hagan et al., 2022), possibly due to breed- or management-related differences.

### 4.2 Model selection

The model comparison results underscore the importance of maternal effects alongside genetic influences on growth and efficiency in MG goats, consistent with earlier studies (Besufkad et al., 2024; Patil et al., 2024; Rahim et al., 2025). The clear superiority of Model 1, which simultaneously included AAG, SLAG, MGE, and MPE effects, demonstrates that omitting any of these components leads to a significant reduction in model fit. This aligns with the conclusions of Ghafouri-Kesbi and Abbasi (2019) and Mohammadi et al. (2019), who emphasized the essential role of maternal environmental effects in explaining pre-weaning growth traits. Similarly, Ghafouri-Kesbi and Eskandarinasab (2024) reported that inclusion of SLAG effects in models already incorporating direct and maternal components markedly improved the fit of efficiency traits in Zandi sheep, as evidenced by a significant reduction in AIC values.

Notably, the exclusion of maternal effects (Model 3) resulted in a more severe degradation in model fit (ΔAIC = +47.7) than the omission of sex-linked effects (Model 2, ΔAIC = +28.9), suggesting that maternal influences collectively exert a stronger impact on these traits than do sex-linked additive genetic components. This observation is consistent with the work of Latifi et al. (2020) in Markhoz goats, who also reported a more pronounced maternal than sex-linked influence in similar traits. At the same time, the significant AIC penalty incurred when sex-linked additive genetic effects were excluded confirms that X-chromosomal additive variation, though modest compared to autosomal and maternal sources, still contributes meaningfully to trait variation. This supports the growing recognition that ignoring sex-linked inheritance can lead to biased genetic parameter estimates, as highlighted in recent genomic studies.

Furthermore, the severe overparameterization observed in Model 4, which included a direct–maternal genetic covariance, underscores the statistical imprecision and numerical instability associated with estimating Cov(a,m) in this population. The pronounced AIC penalty (ΔAIC > 210) indicates that the data lack the necessary informational structure to support such a complex parameterization. This result resonates with reports in other livestock species, where improper modeling of direct–maternal covariance has been shown to inflate standard errors and reduce model reliability.

### 4.3 Maternal effects

Maternal effects are major contributors to phenotypic variation in early growth traits of Murciano-Granadina goats and should be considered in genetic evaluation models alongside direct additive effects. Several studies have stressed the necessity of incorporating maternal components for preweaning growth traits (Rout et al., 2018; Wang et al., 2024). Omitting these effects can bias direct heritability estimates, and both additive and maternal effects have been identified as sources of environmental variation (Tesema et al., 2020). The values of $h_{m}^{2}$ declined with age, from 0.13 for BWT to 0.05 for WWT, consistent with the notion that maternal influences diminish as dependency on the dam decreases (Kashi et al., 2022). This result was in agreement with reports of Latifi et al. (2020), who stated that maternal effect tends to diminish at later ages. In the present study, MPE effects were important for the genetic evaluation of BW in Murciano-Granadina goats. Rout et al. (2018) similarly indicated that MPE effects are more influential in earlier age (e.g., BWT) and gradually decline thereafter.

The necessity of including both MGE and MPE effects for accurate evaluation of early growth traits in the same goat population was also emphasized by Mokhtari et al. (2024). Overall, the influence of maternal effects on growth and efficiency-related traits has been widely documented across goat breeds such as Raeini Cashmere (Mohammadi et al., 2012), Barbari (Singh et al., 2022), Markhoz (Latifi et al., 2020), Jamunapari (Dige et al., 2021), and Inner Mongolia White Arbas Cashmere goats (Wang et al., 2023). These findings collectively underscore the importance of incorporating both MGE and MPE components into the genetic evaluation of early-growth traits. These results, highlights the need to include MTG and MPE effects in the genetic evaluation of early-growth traits.

In addition to maternal effects, a portion of the additive genetic variance in growth traits may result from maternal genomic imprinting, where gene expression depends on parent-of-origin (Ehsaninia & Ghafouri-Kesbi, 2024). While not explicitly modeled here, acknowledging maternal imprinting helps contextualize maternal contributions and highlights the need for future analyses incorporating these effects.

### 4.4 Autosomal additive genetic effects

The values of $h_{a}^{2}$ for BWT and WWT increased with age, suggesting stronger additive genetic influence at later stages, possibly due to gene expression changes or reduced data from preweaning mortality and culling. Ehsaninia and Ghafouri-Kesbi (2024) reported similarly low $h_{a}^{2}$ for growth and efficiency traits (0.05–0.11), as did Mokhtari et al. (2024) (0.04–0.08). The minor differences between our estimates and these earlier studies likely reflect our more comprehensive model specification that included sex-linked genetic effects, leading to more accurate partitioning of variance components. Similar findings have been reported in West African Dwarf (Ofori & Hagan, 2020) and Turkish Saanen goats (Ataç et al., 2023). Overall, $h_{a}^{2}$ values were low (0.05–0.09), with BWT showing the lowest value, consistent with findings in Beetal (Magotra et al., 2018) and Turkish Saanen (Ataç et al., 2023) goats. However, higher estimates have been observed in Boer × Central Highland (Tesema et al., 2021) and Salem Black (Patil et al., 2024) goats, while extremely low values were reported in Osmanabadi (Jedhe et al., 2009) goats. For WWT ($h_{a}^{2}$ = 0.08), the result aligns with estimates in Markhoz (Latifi et al., 2020) and Salem Black (Patil et al., 2024) goats but contrasts with higher values in Jamunapari (Rout et al., 2018) and Black Bengal (Jasmine & Sanjoy, 2022), and lower ones in Markhoz (Rashidi et al., 2011).

The $h_{a}^{2}$ for PWGR was 0.07, consistent with reports for Boer (Zhang et al., 2009), Sirohi (Gowane et al., 2011), and Raeini Cashmere (Barazandeh et al., 2012) goats, but lower than values reported for Jamunapari (Dige et al., 2021), Mehsana (Gupta et al., 2016), and Mongolian Arbas (Wang et al., 2023) goats. Among the efficiency traits, PWKR had the highest $h_{a}^{2}$ (0.09), exceeding estimates in Jakhrana (Bangar et al., 2020) and Raeini Cashmere (Barazandeh et al., 2012) and, but lower than those for Boer × Central Highland (Tesema et al., 2020), Beetal (Magotra et al., 2018), and Markhoz (Naderi & Latifi, 2023) goats. Variations in $h_{a}^{2}$ may result from differences in breed, environment, data structure, or model specification. Although these traits exhibit low heritability and limited response to selection, their economic relevance justifies efforts for genetic improvement.

### 4.5 X-linked additive genetic effects

Our findings indicate that X-linked genetic effects account for approximately 1–4 % of the variation in growth- and efficiency-related traits. The sex-linked heritability ($h_{s}^{2}$) declined from 0.03 for BWT to 0.02 for WWT, suggesting a limited role of X-linked genes in early growth. These estimates are consistent with previous findings in Markhoz goats (Latifi et al., 2020; Naderi & Latifi, 2023). Across species, $h_{s}^{2}$ values for efficiency traits vary widely, from 0.00 in Baluchi sheep (Noorian et al., 2020) to 0.14 in Lori-Bakhtiari sheep (Maraveni et al., 2018). In our study, when SLAG effects were excluded from the model, both autosomal additive and residual variances increased by 3–14 % and 7–20 %, respectively, indicating a substantial redistribution of variance components when this source of variation is ignored. This trend aligns with findings from Zandi sheep, where accounting for X-linked inheritance reduced $\sigma_{e}^{2}$ by 7–19 % and $\sigma_{a}^{2}$ by around 10 % (Ghafouri-Kesbi & Eskandarinasab, 2024). Similarly, Noorian et al. (2020) observed a 20 % decline in $\sigma_{s}^{2}$ for efficiency traits in Baluchi sheep after including SLAG effects.

Improved accuracy in genetic evaluation when X-linked effects are included has also been reported (Ghafouri-Kesbi & Abbasi, 2019; Ghafouri Kesbi et al., 2024). For PWGE, while goat-specific $h_{s}^{2}$ estimates are lacking, previous studies in Markhoz goats revealed $h_{s}^{2}$ values ranging from 0.00 (Latifi et al., 2017) to 0.02 (Naderi & Latifi, 2023) for PWGR. In sheep, $h_{s}^{2}$ estimates for PWGR reached 0.07 in Mehraban (Latifi, 2018), whereas near-zero values for PWKR and PWGE were observed in Baluchi (Noorian et al., 2020) and higher values (0.04 for PWKR) in Zandi sheep (Ghafouri Kesbi et al., 2024), suggesting potential breed-specific effects. These findings suggest that X-linked genes play a small or negligible role in the genetic improvement of preweaning performance in goats. Collectively, these consistent results confirm that genes on the X chromosome contribute meaningfully to phenotypic variation in growth and efficiency traits, and that properly modeling X-linked inheritance enhances model accuracy by preventing the misallocation of genetic variance to other components.

Although the estimated X-linked additive variance was modest (≈1–4 %), part of this variation could arise from epigenetic regulation rather than strictly Mendelian inheritance. Mechanisms such as genomic imprinting and X-chromosome dosage compensation can produce parent-of-origin or sex-specific expression patterns that may be partially captured as X-linked variance in pedigree-based models (Ghafouri-Kesbi & Eskandarinasab, 2024). Previous studies in goats and sheep have generally reported small or inconsistent imprinting effects on growth traits (Amiri Roudbar et al., 2017; 2018; Mokhtari et al., 2022). While not explicitly modeled here, acknowledging these mechanisms helps contextualize the observed X-linked variance and supports the view that both genetic and epigenetic processes shape growth-related traits in small ruminants.

### 4.6 Genetic correlations: autosomal vs X-linked

Genetic correlations reflect shared polygenic architecture between traits. While X-linked additive genetic correlations (r_s_) for growth- and efficiency traits have not been previously reported in goats, the estimates of r_a_ and r_s_ in this study were similar to those reported by Latifi et al. (2020), Patile et al. (2024) and Magotra et al. (2018). Positive r_a_ and r_s_ values indicate shared genetic influences via autosomal or X-linked loci. Snell and Turner (2018) suggested that such positive r_s_ values may result from shared regulatory mechanisms on the X chromosome, including hormonal pathways like the IGF-1 axis, epigenetic regulation, or dosage compensation. Strong r_a_ and r_s_ between PWGR and PWKR imply common genetic control, possibly due to pleiotropy or linkage, supporting the feasibility of improving both traits simultaneously. These findings indicate that animals with higher PWGRs are also efficient users of feed and vice versa and that positive r_a_ and r_s_ values between traits guarantee the success of multi-trait selection programs, including growth rate and efficiency-related traits. Conversely, negative r_s_ values between BWT and PWGR or PWGE suggest that shared X-linked genes may have antagonistic effects on these traits.

### 4.7 Genetic trends in EBVs: autosomal vs X-linked

Autosomal genetic trends observed in this study were generally low but variable across early growth traits. Positive autosomal additive genetic trends for BWT (0.003 kg/year) and WWT (0.005 kg/year), indicating gradual genetic improvement over time, and aligned with previous findings in Jamunapari (Rout et al., 2018) and Markhoz goats (Latifi & Razmkabir, 2019; Hosseinzadeh Shirzeyli et al., 2022), while contrasting with negative trends reported in Boer × Central Highland goats (−0.0805 kg/year) (Tesema et al., 2020). PWGE and PWGR exhibited negative autosomal additive genetic trends, in agreement with Tesema et al. (2024), though other studies have reported slight increases (Bosso et al., 2007; Ghafouri-Kesbi et al., 2009). These variable trends may reflect variability in selection intensity and population structure.

In contrast, X-linked EBVs showed generally weak and trait-dependent trends, with slight negative slopes for BWT, WWT, and PWGE, but marginally positive values for PWGR and PWKR. This pattern indicates that the contribution of X-linked loci to temporal genetic change is limited and variable across traits, likely reflecting inconsistent selection pressure on sex-linked genes. In BLUP-based animal models, estimated breeding values are derived from an individual’s own performance, pedigree, and records of relatives. Consequently, the negative trends in early growth traits may reflect weak or inconsistent selection pressure, particularly the lack of EBV-based selection for dams. The hemizygous nature of the X chromosome in males reduces genetic variance and limits the response to selection for X-linked loci (Carvalheiro et al., 2014; Uemoto et al., 2019). Furthermore, X-linked inheritance results in sex-specific expression patterns, complicating the accurate estimation and effective use of X-linked genetic effects (Wu et al., 2018). Breeding programs typically focus on autosomal selection, which may unintentionally overlook or even select against beneficial X-linked alleles due to pleiotropic effects or negative associations with efficiency-related traits (García-Ruiz et al., 2016). In addition, inadequate modeling of maternal and sex-linked additive genetic effects can contribute to biased or underestimated X-linked EBVs (Zhang et al., 2021).

The present results provide new insights into the genetic architecture of growth and efficiency traits; however, several limitations must be acknowledged. First, the relatively shallow pedigree, encompassing a limited number of generations, may diminish the precision of genetic parameter estimates, particularly concerning low-frequency alleles and subtle X-linked effects. Second, reliance on pedigree-based relationships, rather than high-density genomic information, restricts the capacity to capture Mendelian sampling variance and may result in an underestimation of contributions from both X-linked and non-additive loci. Third, the results may be sensitive to model specification, including the selection of fixed effects, treatment of (co)variances, and the assumed variance–covariance structures. Although model selection was informed by statistical criteria, these assumptions could influence the magnitude and significance of the estimated autosomal additive, sex-linked additive, and maternal effects. Recognizing these limitations enhances transparency, contextualizes the interpretation of effect sizes, and delineates the scope for generalizing the findings to breeding applications.

## 5 Conclusion

This study provides a comprehensive partitioning of genetic variance into autosomal and X-linked components for preweaning growth- and efficiency traits in Murciano-Granadina goats. The results highlight that maternal effects have a substantial influence on early growth, and their inclusion in genetic evaluation models is essential to avoid overestimating direct heritability. Autosomal additive genetic effects showed a modest but consistent contribution to trait variation, while X-linked effects explained a minor proportion of the total variance (1–4 %), with low sex-linked heritability estimates. Genetic correlations between growth and efficiency traits, especially between PWGR and PWKR, revealed shared genetic control and supported the potential for multi-trait selection. Despite generally low autosomal genetic trends, some efficiency traits showed favorable improvement over time. In contrast, the contribution of X-linked loci to overall genetic progress appears limited, likely due to the hemizygous nature of the X chromosome, sex-specific gene expression, and limited selection pressure on sex-linked loci. These findings emphasize the necessity of incorporating maternal and sex-linked additive genetic effects into genetic models to enhance the accuracy of breeding value predictions and to inform selection strategies aimed at improving early growth and efficiency traits in Murciano-Granadina goats.

## Funding

This research did not receive any specific grant from funding agencies in the public, commercial, or not-for-profit sectors.

## Data availability

The data that support the findings of this study are available from a private farm but restrictions apply to the availability of these data, which were used under license for the current study, and so are not publicly available. Data are however available from the corresponding author upon reasonable request and with permission and with permission from the data provider.

## Ethical approval

This study did not involve any experimental procedures on live animals. All data used were derived from routinely collected performance and pedigree records from a commercial goat breeding farm.

## CRediT authorship contribution statement

**Jamshid Ehsaninia:** Writing – review & editing, Writing – original draft, Methodology, Formal analysis, Conceptualization. **Mohammad Bagher Zandi:** Writing – review & editing, Validation, Methodology, Investigation. **Moein Taned:** Software, Formal analysis. **Alireza Bagheripour:** Conceptualization.

## Declaration of competing interest

The authors declare that they have no known competing financial interests or personal relationships that could have appeared to influence the work reported in this paper.

## Appendix Supplementary materials

Image, application 1

Image, application 2
