# Supplementary material for: Deciphering autosomal and X-linked genetic effects of early growth traits in Murciano-Granadina goats via a multivariate animal model
Source: Vet Anim Sci. 2025 Dec 7;31:100553. doi: 10.1016/j.vas.2025.100553 (PMC12756649; doi:10.1016/j.vas.2025.100553)
Supplement: Supplementary file 1 [file mmc1.docx]

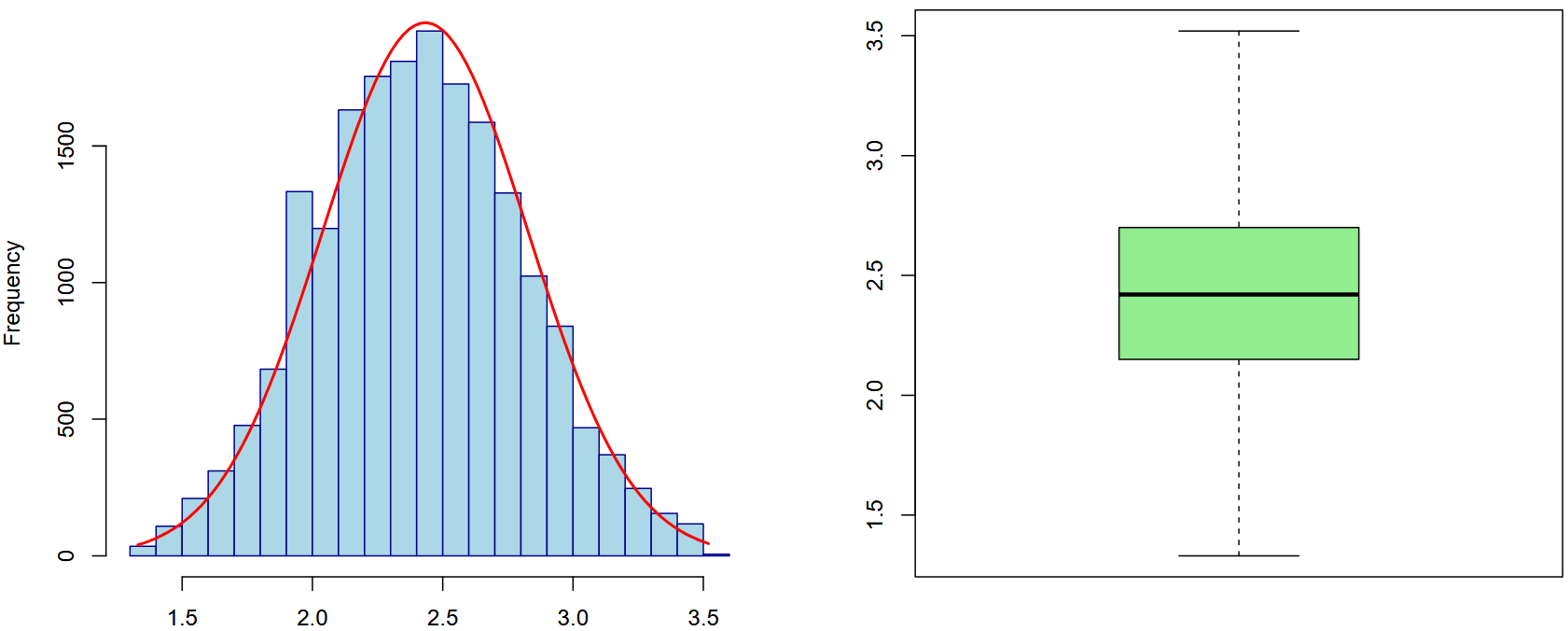


**Supplementary Figure S1.** Histogram and boxplot of birth weight (BWT) in Murciano-Granadina goats.


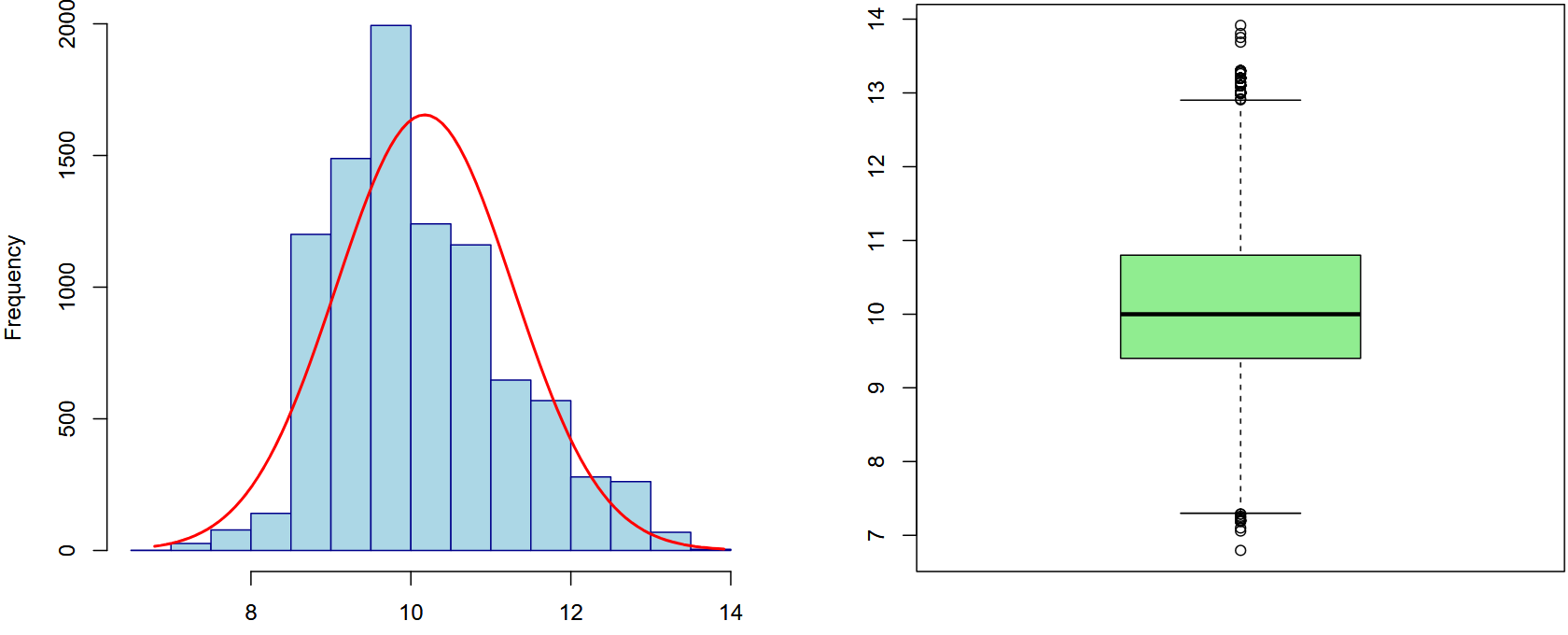


**Supplementary Figure S2.** Histogram and boxplot of weaning weight (WWT) in Murciano-Granadina goats.


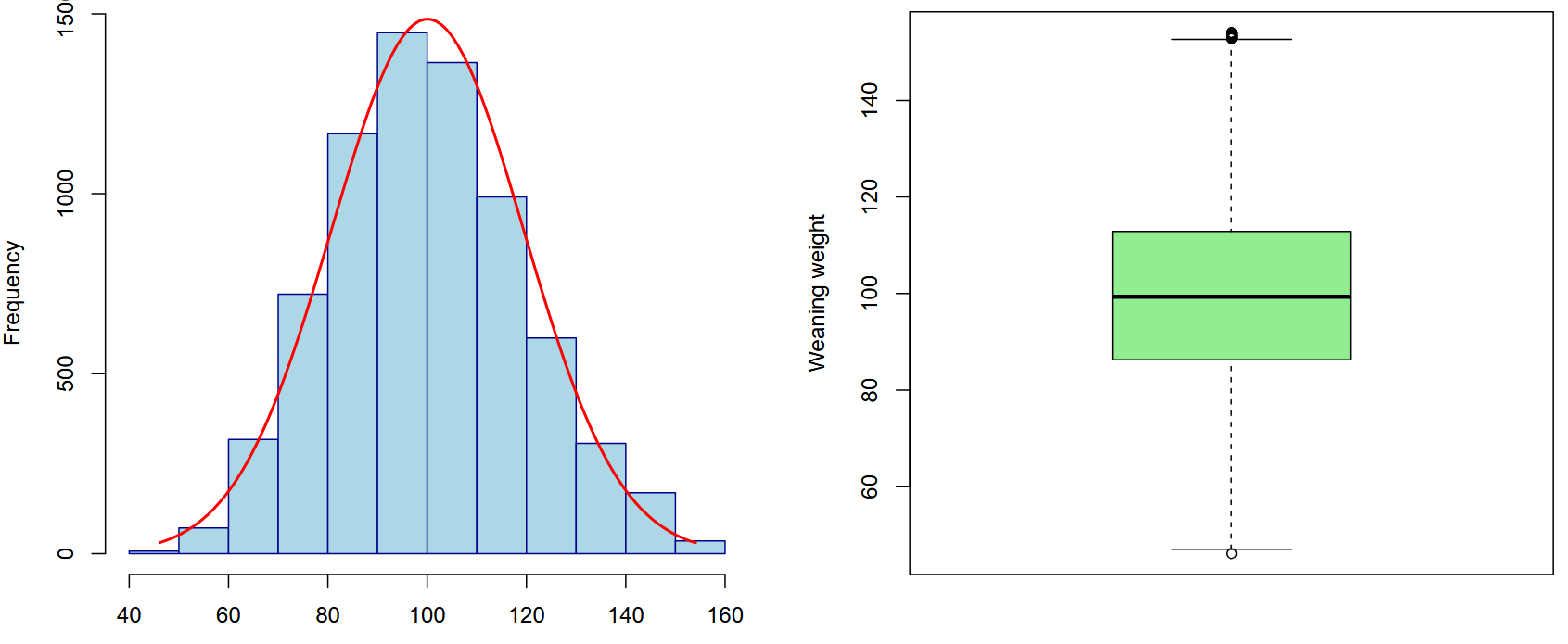


**Supplementary Figure S3.** Histogram and boxplot of pre-weaning growth rate (PWGR) in Murciano-Granadina goats.


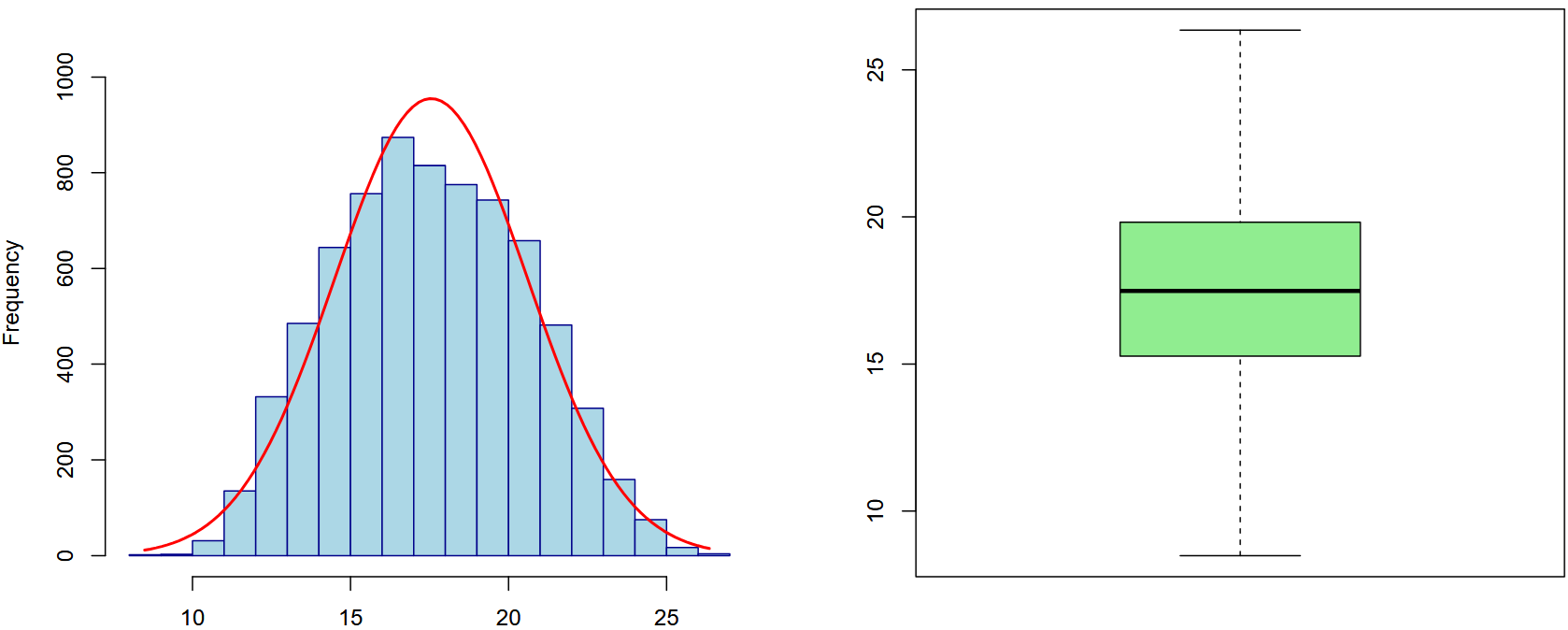


**Supplementary Figure S4.** Histogram and boxplot of pre-weaning Klieber ratio (PWKR) in Murciano-Granadina goats.


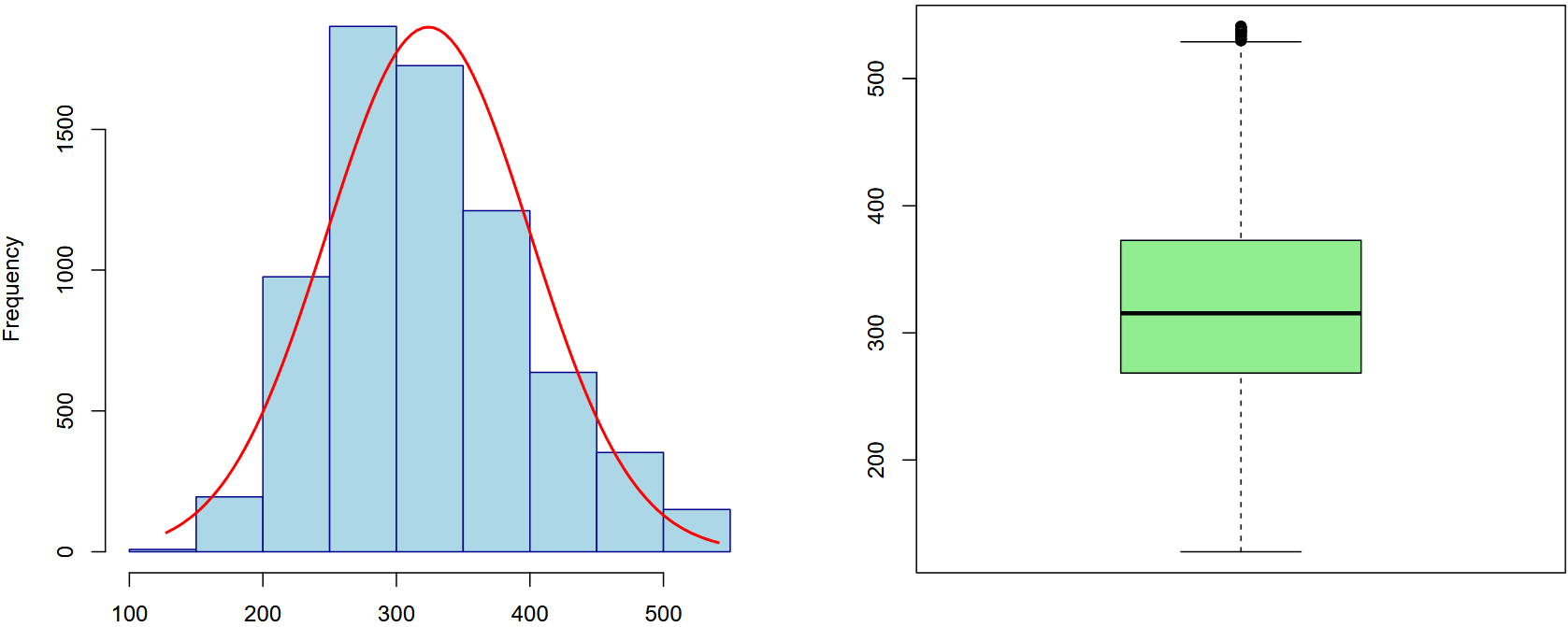


**Supplementary Figure S5.** Histogram and boxplot of pre-weaning gain efficiency (PWGE) in Murciano-Granadina goats.
