# Supplementary material for: Deciphering autosomal and X-linked genetic effects of early growth traits in Murciano-Granadina goats via a multivariate animal model
Source: Vet Anim Sci. 2025 Dec 7;31:100553. doi: 10.1016/j.vas.2025.100553 (PMC12756649; doi:10.1016/j.vas.2025.100553)
Supplement: Supplementary file 2 [file mmc2.docx]

| **Supplementary Table S1**  Estimates of variance components and genetic parameters for birth weight (BWT) and weaning weight (WWT) in Murciano-Granadina goats (best model in bold). | | | | | | | | | | | | | | |
| --- | --- | --- | --- | --- | --- | --- | --- | --- | --- | --- | --- | --- | --- | --- |
| Trait | Model | $\sigma_{a}^{2}$ | $\sigma_{s}^{2}$ | $\sigma_{\mathrm{pe}}^{2}$ | $\sigma_{m}^{2}$ | $\sigma_{\mathrm{am}}$ | $\sigma_{e}^{2}$ | $\sigma_{p}^{2}$ | $h_{a}^{2}$ ± SE | $h_{s}^{2}$ ± SE | $\mathrm{pe}^{2}$ ± SE | $h_{m}^{2}$ ± SE | r_am_ ± SE | AIC |
| BWT | 1 | 0.041 | 0.003 |  |  |  | 0.097 | 0.141 | 0.29 ± 0.02 | 0.02 ± 0.01 |  |  |  | -15,457.21 |
|  | 2 | 0.006 | 0.001 | 0.020 |  |  | 0.107 | 0.134 | 0.04 ± 0.01 | 0.01 ± 0.01 | 0.15 ± 0.01 |  |  | -15,679.92 |
|  | 3 | 0.007 | 0.002 |  | 0.016 |  | 0.111 | 0.135 | 0.05 ± 0.01 | 0.01 ± 0.01 |  | 0.12 ± 0.01 |  | -15,596.20 |
|  | 4 | 0.006 | 0.001 |  | 0.022 | -0.004 | 0.109 | 0.134 | 0.04 ± 0.01 | 0.02 ± 0.01 |  | 0.16 ± 0.02 | -0.37 ± 0.17 | -15,639.95 |
|  | **5** | **0.005** | **0.001** | **0.016** | **0.004** |  | **0.106** | **0.137** | **0.04 ± 0.01** | **0.01 ± 0.01** | **0.12 ± 0.01** | **0.03 ± 0.01** |  | **-15,682.24** |
|  | 6 | 0.006 | 0.001 | 0.016 | 0.006 | -0.003 | 0.107 | 0.134 | 0.04 ± 0.01 | 0.01 ± 0.01 | 0.12 ± 0.01 | 0.05 ± 0.02 | -0.49 ± 0.23 | -15,680.08 |
| WWT | 1 | 0.166 | 0.011 |  |  |  | 1.327 | 1.504 | 0.11 ± 0.02 | 0.01 ± 0.01 |  |  |  | 10,326.07 |
|  | 2 | 0.105 | 0.001 | 0.087 |  |  | 1.302 | 1.500 | 0.11 ± 0.02 | 0.01 ± 0.02 | 0.06 ± 0.01 |  |  | 10,311.00 |
|  | **3** | **0.107** | **0.001** |  | **0.071** |  | **1.317** | **1.496** | **0.07 ± 0.02** | **0.01 ± 0.01** |  | **0.05 ± 0.01** |  | **10,306.49** |
|  | 4 | 0.144 | 0.006 |  | 0.174 | -0.116 | 1.291 | 1.500 | 0.10 ± 0.03 | **0.01 ± 0.01** | 0.11 ± 0.01 | 0.12 ± 0.03 | -0.73± 0.16 | 10,308.69 |
|  | 5 | 0.105 | 0.001 | 0.086 | 0.001 |  | 0.039 | 0.136 | 0.07 ± 0.02 | 0.01 ± 0.01 | 0.06 ± 0.02 | 0.01 ± 0.02 |  | 10,313.00 |
|  | 6 | 0.142 | 0.007 | 0.076 | 0.092 | -0.097 | 1.278 | 1.500 | 0.10 ± 0.03 | 0.07 ± 0.02 | 0.05 ± 0.03 | 0.06 ± 0.04 | -0.85 ± 0.19 | 10,315.80 |
| $\sigma_{a}^{2}$: additive genetic variance for autosomal loci, $\sigma_{s}^{2}$: additive genetic variance for sex-linked loci, $\sigma_{\mathrm{pe}}^{2}$: maternal permanent environmental variance, $\sigma_{m}^{2}$: maternal additive genetic variance, $\sigma_{a,m}$: direct-maternal additive genetic covariance, $\sigma_{e}^{2}$: residual variance, $\sigma_{P}^{2}$: phenotypic variance, $h_{a}^{2}$: direct autosomal heritability, $h_{s}^{2}$: direct sex-linked heritability, $\mathrm{pe}^{2}$: maternal permanent environmental effect, $h_{m}^{2}$: maternal heritability, $r_{a,m}$: direct-maternal additive genetic correlation, SE: standard error. | | | | | | | | | | | | | | |

| **Supplementary Table S2**  Estimates of variance components and genetic parameters for preweaning growth rate (PWGR) and preweaning growth efficiency (PWGE) in Murciano-Granadina goats (best model in bold). | | | | | | | | | | | | | | |
| --- | --- | --- | --- | --- | --- | --- | --- | --- | --- | --- | --- | --- | --- | --- |
| Trait | Model | $\sigma_{a}^{2}$ | $\sigma_{s}^{2}$ | $\sigma_{\mathrm{pe}}^{2}$ | $\sigma_{m}^{2}$ | $\sigma_{\mathrm{am}}$ | $\sigma_{e}^{2}$ | $\sigma_{p}^{2}$ | $h_{a}^{2}$ ± SE | $h_{s}^{2}$ ± SE | $\mathrm{pe}^{2}$ ± SE | $h_{m}^{2}$ ± SE | r_am_ ± SE | AIC |
| PWGR | 1 | 40.00 | 5.94 |  |  |  | 324.47 | 370.41 | 0.11 ± 0.02 | 0.02 ± 0.01 |  |  |  | 50,340.14 |
|  | 2 | 24.80 | 1.69 | 20.59 |  |  | 320.52 | 367.60 | 0.07 ± 0.02 | 0.01 ± 0.01 | 0.06 ± 0.01 |  |  | 50,325.00 |
|  | **3** | **24.98** | **0.001** |  | **19.15** |  | **323.27** | **367.40** | **0.07 ± 0.02** | **0.00 ± 0.01** |  | **0.05 ± 0.01** |  | **50,318.76** |
|  | 4 | 26.68 | 0.837 |  | 38.67 | -19.19 | 320.24 | 367.24 | 0.07 ± 0.02 | 0.02 ± 0.01 |  | 0.11 ± 0.03 | -0.60 ± 0.20 | 50,323.97 |
|  | 5 | 23.60 | 0.005 | 4.80 | 16.88 |  | 321.85 | 367.12 | 0.06 ± 0.02 | 0.00 ± 0.01 |  | 0.05 ± 0.03 |  | 50,323.86 |
|  | 6 | 26.722 | 1.02 | 5.39 | 33.39 | -18.73 | 319.47 | 367.26 | 0.07 ± 0.02 | 0.00 ± 0.01 | 0.02 ± 0.03 | 0.09± 0.04 | -0.63 ± 0.21 | 50,320.76 |
| PWGE | 1 | 662.33 | 346.78 |  |  |  | 5821.10 | 6830.20 | 0.10 ± 0.03 | 0.05 ± 0.02 |  |  |  | 71,457.17 |
|  | 2 | 197.79 | 167.40 | 672.52 |  |  | 5692.8 | 6730.6 | 0.03 ± 0.02 | 0.03 ± 0.01 | 0.10 ± 0.01 |  |  | 71,408.06 |
|  | **3** | **188.96** | **136.04** |  | **630.64** |  | **5768.20** | **6723.83** | **0.03 ± 0.01** | **0.02 ± 0.01** |  | **0.09 ± 0.01** |  | **71,402.57** |
|  | 4 | 242.09 | 192.16 |  | 977.13 | -0.116 | 5708.33 | 6738.66 | 0.04 ± 0.01 | **0.03 ± 0.01** |  | 0.15 ± 0.03 | -0.78 ± 0.26 | 71,404.61 |
|  | 5 | 187.34 | 388.99 | 142.45 | 294.78 |  | 5709.89 | 6723.44 | 0.03 ± 0.02 | 0.02 ± 0.01 | 0.06 ± 0.03 | 0.04 ± 0.02 |  | 71,407.03 |
|  | 6 | 234.50 | 208.36 | 446.67 | 564.90 | -362.41 | 5649.00 | 6741.02 | 0.04 ± 0.02 | 0.03 ± 0.01 | 0.07 ± 0.03 | 0.08 ± 0.04 | -0.99 ± 0.32 | 71,408.65 |
| $\sigma_{a}^{2}$: additive genetic variance for autosomal loci, $\sigma_{s}^{2}$: additive genetic variance for sex-linked loci, $\sigma_{\mathrm{pe}}^{2}$: maternal permanent environmental variance, $\sigma_{m}^{2}$: maternal additive genetic variance, $\sigma_{a,m}$: direct-maternal additive genetic covariance, $\sigma_{e}^{2}$: residual variance, $\sigma_{P}^{2}$: phenotypic variance, $h_{a}^{2}$: direct autosomal heritability, $h_{s}^{2}$: direct sex-linked heritability, $\mathrm{pe}^{2}$: maternal permanent environmental effect, $h_{m}^{2}$: maternal heritability, $r_{a,m}$: direct-maternal additive genetic correlation, SE: standard error. | | | | | | | | | | | | | | |

| **Supplementary Table S3**  Estimates of variance components and genetic parameters for preweaning Klieber ratio (PWKR) in Murciano-Granadina goats (best model in bold). | | | | | | | | | | | | | | |
| --- | --- | --- | --- | --- | --- | --- | --- | --- | --- | --- | --- | --- | --- | --- |
| Trait | Model | $\sigma_{a}^{2}$ | $\sigma_{s}^{2}$ | $\sigma_{\mathrm{pe}}^{2}$ | $\sigma_{m}^{2}$ | $\sigma_{\mathrm{am}}$ | $\sigma_{e}^{2}$ | $\sigma_{p}^{2}$ | $h_{a}^{2}$ ± SE | $h_{s}^{2}$ ± SE | $\mathrm{pe}^{2}$ ± SE | $h_{m}^{2}$ ± SE | r_am_ ± SE | AIC |
| PWKR | 1 | 1.00 | 0.165 |  |  |  | 6.70 | 7.87 | 0.13 ± 0.02 | 0.02 ± 0.01 |  |  |  | 22,272.16 |
|  | 2 | 0.711 | 0.092 | 0.358 |  |  | 6.65 | 7.81 | 0.09 ± 0.02 | 0.01 ± 0.01 | 0.06 ± 0.01 |  |  | 22,262.94 |
|  | **3** | **0.724** | **0.031** |  | **0.341** |  | **6.70** | **7.80** | **0.09 ± 0.02** | **0.00 ± 0.01** |  | **0.04 ± 0.01** |  | **22,253.96** |
|  | 4 | 0.842 | 0.055 |  | 0.836 | -0.538 | 6.61 | 7.80 | 0.11 ± 0.3 | 0.01 ± 0.01 |  | 0.11 ± 0.03 | -0.64 ± 0.16 | 22,259.32 |
|  | 5 | 0.690 | 0.043 | 0.075 | 0.305 |  | 6.68 | 7.79 | 0.09 ± 0.02 | 0.00 ± 0.01 | 0.01 ± 0.02 | 0.04 ± 0.02 |  | 22,262.03 |
|  | 6 | 0.840 | 0.064 | 0.194 | 0.650 | -0.528 | 6.58 | 7.80 | 0.11 ± 0.03 | 0.00 ± 0.01 | 0.03 ± 0.03 | 0.08± 0.04 | -0.71 ± 0.19 | 22,254.74 |
| $\sigma_{a}^{2}$: additive genetic variance for autosomal loci, $\sigma_{s}^{2}$: additive genetic variance for sex-linked loci, $\sigma_{\mathrm{pe}}^{2}$: maternal permanent environmental variance, $\sigma_{m}^{2}$: maternal additive genetic variance, $\sigma_{a,m}$: direct-maternal additive genetic covariance, $\sigma_{e}^{2}$: residual variance, $\sigma_{P}^{2}$: phenotypic variance, $h_{a}^{2}$: direct autosomal heritability, $h_{s}^{2}$: direct sex-linked heritability, $\mathrm{pe}^{2}$: maternal permanent environmental effect, $h_{m}^{2}$: maternal heritability, $r_{a,m}$: direct-maternal additive genetic correlation, SE: standard error. | | | | | | | | | | | | | | |

| **Supplementary Table S4**  Estimates of autosomal and sex-linked of estimated breeding values (EBVs) for pre-weaning growth traits in Murciano-Granadina goats. | | | | | | | | | |
| --- | --- | --- | --- | --- | --- | --- | --- | --- | --- |
| Trait | Direct genetic autosomal EBVs | | | |  | Direct genetic sex-linked EBVs | | | |
|  | Mean | SD | Minimum | Maximum |  | Mean | SD | Minimum | Maximum |
| BWT | 0.003 | 0.03 | -0.18 | 0.11 |  | 0.002 | 0.02 | -0.09 | 0.10 |
| WWT | 0.012 | 0.16 | -0.46 | 1.20 |  | -0.01 | 0.06 | -0.32 | 0.28 |
| PWGR | 0.050 | 2.03 | -5.91 | 21.19 |  | -0.12 | 0.85 | -4.29 | 3.05 |
| PWKR | 0.001 | 0.40 | -1.34 | 1.77 |  | -0.01 | 0.09 | -0.49 | 0.27 |
| PWGE | 0.79 | 9.09 | -23.97 | 88.88 |  | -0.75 | 6.01 | -28.82 | 23.15 |
| BWT: birth weight, WWT: weaning weight, PWGR: preweaning growth rate, PWKR: preweaning Kleiber ratio, PWGE: preweaning growth efficiency, SD: standard deviation. | | | | | | | | | |
